# A short-term manual for webcam-based telemedicine treatment of Internet use disorders

**DOI:** 10.3389/fpsyt.2023.1053930

**Published:** 2023-02-23

**Authors:** Magdalena Pape, Birte Linny Geisler, Lorraine Cornelsen, Laura Bottel, Bert Theodor te Wildt, Michael Dreier, Stephan Herpertz, Jan Dieris-Hirche

**Affiliations:** ^1^Department of Psychosomatic Medicine and Psychotherapy, LWL-University Hospital, Ruhr University Bochum, Bochum, Germany; ^2^Psychosomatic Hospital Diessen Monastery, Diessen am Ammersee, Germany; ^3^Department of Psychosomatic Medicine and Psychotherapy, University Medical Center Mainz, Johannes Gutenberg University Mainz, Mainz, Germany

**Keywords:** Internet use disorder, Internet-based treatment, online therapy, Internet-based intervention, treatment manual, telemedicine, eHealth, gaming disorder

## Abstract

In recent decades, the number of people who experience their Internet use behavior as problematic has risen dramatically. In Germany, a representative study from 2013 estimated the prevalence of Internet use disorder (IUD) to be about 1.0%, with higher rates among younger people. A 2020 meta-analysis shows a global weighted average prevalence of 7.02%. This indicates that developing effective IUD treatment programs is more critical than ever. Studies show that motivational interviewing (MI) techniques are widely used and effective in treating substance abuse and IUDs. In addition, an increasing number of online-based health interventions are being developed to provide a low-threshold treatment option. This article presents a short-term online-based treatment manual for IUDs that combines MI techniques with therapy tools from cognitive behavioral therapy (CBT) and acceptance and commitment therapy (ACT). The manual includes 12 webcam-based therapy sessions, each lasting 50 min. Each session is framed by a standardized beginning, conclusion, outlook, and flexible session content. In addition, the manual contains example sessions to illustrate the therapeutic intervention. Finally, we discuss the advantages and disadvantages of online-based therapy compared to analog treatment settings and provide recommendations for dealing with these challenges. By combining established therapeutic approaches with an online-based therapeutic setting based on flexibility and motivation, we aim to provide a low-threshold solution for treating IUDs.

## 1. Introduction

### 1.1. Internet use disorders

In 2021, around 88% of the German population (>14 years) used the Internet at least irregularly (1). The average daily usage time was 136 min, a 16-min increase compared to 2020 (2). The number of people who perceive their Internet use behavior as problematic has increased dramatically in recent years, in part because of the social restrictions imposed by the COVID-19 pandemic (3, 4). Internet use disorder (IUD) is an umbrella term encompassing excessive behaviors of various Internet patterns, such as gaming, pornography, shopping, social media, or video streaming. With the introduction of the 11th revision of the International Classification of Diseases (ICD-11) published by the World Health Organization (WHO), IUDs can be coded as “disorders due to addictive behaviors” (5).

To date, only gambling (code: 6C50) and gaming disorder (code: 6C51) have been defined as standalone disorders. In contrast, other IUD subtypes must be coded as “other specified disorders due to addictive behaviors” (code: 6C5Y). IUDs are characterized by (a) impaired control over usage behavior, (b) preoccupation with and prioritization of usage behavior over previously enjoyable or essential activities, and (c) escalation of use despite negative consequences, such as loss of relationships or employment, all lasting at least 12 months. Predisposing factors differ between subtypes of IUD (6). For example, young men with poor academic performance (7), who exhibit procrastination (8) and deficits in emotional awareness and processing (9), are particularly at risk for developing gaming disorders. Social network use disorder (SNUD) is associated with the female gender (10), higher perceived social loneliness, the need to belong, and the fear of missing out (11). Excessive pornography use is more common in men with heightened sexual motivation (12) and an anxious partner attachment style (13), many of whom experienced negative life events in early childhood (14). In a representative study, the prevalence of IUD in Germany was estimated at 1.0%, with increased rates (4–8.4%) in adolescents (15, 16). A 2020 meta-analysis found a global average prevalence of 7.02% (17). Those affected often suffer from comorbid mental disorders, e.g., depression, anxiety disorders, or attention deficit hyperactivity disorder (ADHD) (18–20). IUD patients, i.e., those who have comorbid mental conditions, experience a poor quality of life (QoL) (21) and exhibit increased suicidal symptoms (22). The variability in risk factors and severity of comorbid disorders highlights the need for multimodal, integrative treatment approaches tailored to individual patient needs (23, 24).

### 1.2. Online-based treatment of IUDs

Despite its addictive nature, the Internet offers a straightforward and inexpensive communication tool. In recent years, online-based health interventions have been developed worldwide, offering a low-threshold treatment feasible for everyday life and accessible not only in urban areas (25, 26). Online-based health interventions are of particular interest for IUD treatment because of the lack of specialized care centers, e.g., in rural regions. In addition, as reported for other addictive behaviors and substance use disorders (SUDs), affected individuals often exhibit low motivation to engage in behavioral change, underscoring the need for low-threshold interventions (27).

Online-based interventions have been shown to be effective in treating SUDs (28) and behavioral addictions, such as gambling (29). However, only a few studies have examined online-based treatment for IUDs. Most have been pilot studies with small sample sizes and different treatment strategies (30). Between 2016 and 2019, our research group conducted a preliminary study (OASIS) in which two MI-based online counseling sessions were offered to individuals who found their Internet use behavior problematic (27). The study’s main objective was to investigate whether affected individuals could be reached *via* the Internet and referred for conventional medical treatment. Despite the small number of sessions, a significant reduction in IUD severity and time spent online was found. Furthermore, a recently published meta-analysis concluded that online-based IUD treatment could effectively reduce the severity of IUD compared to no-treatment control groups (31).

### 1.3. Lack of systematic approaches

In summary, there is a need for evidence-based, low-threshold IUD therapy, and online-based treatment can play a major role in meeting this growing need. Still, systematic approaches to how effective therapeutic interventions can be delivered online are lacking. To address this gap, we developed a manual for online-based IUD treatment. It not only includes effective therapeutic approaches and tools from traditional therapeutic settings but also considers the opportunities and challenges of an online setting. For example, how can analog IUD interventions be modified for an online-based setting? How can a stable therapeutic relationship be established quickly and sustainably? Our manual focuses on communication techniques and the therapeutic stance. To our knowledge, this is the first published manual for an online-based IUD treatment.

## 2. Materials and equipment

Because combinations of different treatment approaches are considered most effective in treating IUDs (32–34), we developed an online-based short-term treatment manual for IUDs based on MI techniques combined with therapy tools from cognitive behavioral therapy (CBT), acceptance and commitment therapy (ACT), and established IUD treatment manuals.

### 2.1. Evidence of IUD treatment approaches

Behavior change is preceded by the motivation to change, which can be driven by intrinsic (e.g., “I want to live healthier”) or extrinsic reasons (e.g., “My wife wants me to quit gaming”). MI is a patient-centered yet directive approach that aims to strengthen the intrinsic motivation to change. Miller and Rollnick define MI as “a collaborative conversation style for strengthening a person’s motivation and commitment to change” (35). Because the MI technique guides rather than persuades or directs, it is particularly appropriate for people who are still ambivalent or “less ready to change.” For this reason, MI has been shown to be effective in promoting behavior change in lifestyle interventions, resulting, for example, in weight loss (36). It is a widely used and effective intervention technique in SUD and IUD therapy and counseling (32, 37, 38). Specifically, studies indicate effective MI-based treatment for SNUD (39), gaming (40), and gambling disorders (41).

There is evidence of the effectiveness of CBT approaches in reducing the severity of IUD symptoms and time spent online (42–48). In addition, ACT is an area of the CBT approach that targets psychological flexibility (49). Psychological flexibility is a critical skill for initiating and maintaining behavioral changes. A randomized controlled trial (RCT) of a 12-session-based ACT treatment found significant reductions in pornography use compared with a wait-list control condition (50). A recently published systematic review concluded that ACT is an effective tool for treating addiction, although the included studies had methodological limitations (51).

## 3. Methods

The manual was developed as part of the OMPRIS project (DRKS identifier: 00019925), a multi-center, online-based program to reduce problematic Internet use and promote treatment motivation for Internet gaming disorder and use disorders (52). The study was conducted in accordance with the Declaration of Helsinki. Furthermore, the Ruhr-University Bochum Institutional Review Board approved the study (No. 19-6779).

### 3.1. Manual preparation

Experienced therapists and researchers in the field of IUD treatment prepared the manual in the following steps:

1. Review of published German treatment manuals on addiction, behavioral addiction, IUD, and computer game addiction (53–58).
2. Determination of the number and length of sessions in online therapy, considering the question: What is realistic and feasible? The fundamental therapeutic attitudes were especially taken into account. A therapy duration of 12 sessions (short-term therapy in clinical practice) of 50 min each was chosen.
3. Distillation of key interventions, therapeutic tasks, and subject areas. Here, the experience of the experts involved was used.
4. Extension for certain special situations (e.g., procrastination, indebtedness, etc.).
5. Determination of meaningful sequences of interventions (*core sessions*).
6. Presentation and discussion with experts in IUD treatment and research.
7. Presentation of the manual to participating clinics.
8. Incorporation of feedback and corrections.
9. Finalization of the therapy units.

The most helpful and valuable sessions are referred to as *core sessions* in our manual. In addition, intervention sessions on useful individual topics were developed.

### 3.2. Prerequisites

The manual presented here is intended for therapists considering performing webcam-based treatment on IUD patients. On the one hand, webcam-based therapy can be applied to a wide range of patient types; however, there are certain limitations to consider.

Various requirements must be met to offer online therapy as a psychotherapist. Although these guidelines vary from country to country, they include some common points. The patient must give written consent before online therapy begins. In addition, the technology used and electronic data transmission must allow for adequate communication. Online therapy must take place in spaces that offer privacy. Online sessions must be confidential and free of disruptions. The patient and the therapist must be alone in the room. In an emergency (e.g., suicidal tendencies), the patient’s exact name must be identifiable. The patient should be able to identify themselves with an ID card.

The software provider must usually be certified and thus prove that the data protection requirements are guaranteed. As a rule, online therapy must be end-to-end encrypted. Psychotherapists often must also obtain authorization for billing and conducting online therapy. In terms of content, a defined and clear procedure is required at the beginning of treatment in the case of a mental health crisis involving suicidal tendencies. A clear plan must be agreed upon with the patient. For example, it must be clear when the therapist, out of concern, will inform the police or the city’s social psychiatric service and pass on patient data. These regulations should also be signed and accepted in writing by the patient.

Furthermore, it should be discussed which psychiatric contact points near the patient’s home can be visited in an emergency (usually the relevant psychiatric and psychotherapy clinic). Clear rules should also be discussed regarding whether the webcam or only the microphone should be turned on. From experience, we recommend communication *via* the webcam with image and sound.

For a long time, for professional reasons, it was necessary to examine patients in person at least once. However, these guidelines have changed, and the ban on remote treatment has been abolished, making online-based treatments possible. However, if the pathology is very pronounced or there is a limited need for a webcam diagnosis, we still recommend a one-time face-to-face contact to assess the patient’s ability to receive online treatment.

### 3.3. Indication and contraindication

In our experience, the treatment program is suitable for adults and adolescents over 16 years old. Epidemiological studies indicate a higher prevalence of IUDs in adolescents (15). However, adolescents younger than 16 should undergo specific child and youth psychotherapy. The subtype of IUD (e.g., gaming, pornography, and shopping) can vary, as can the severity of the IUD. Webcam-based treatment may be helpful not only for people diagnosed with IUDs but also for at-risk users who may be embarrassed to seek analog therapy while uncertain about their problem’s severity.

Web-based IUD treatment is contraindicated in the presence of coexisting disorders, such as psychotic disorders or major depression with suicidal ideation. In this case, treatment could destabilize the patient and increase psychological distress; it should therefore be administered in a controlled therapeutic environment, such as in an inpatient facility. The therapist should monitor for changes in the patient’s psychological well-being throughout the therapeutic process. If the patient’s condition worsens, the therapist should offer support and, if necessary, refer the patient to a specialized inpatient clinic.

## 4. Results

### 4.1. Therapeutic stance

The therapeutic stance is based on the idea of MI and can be described by four guiding principles, which Rollnick et al. (59) summarized under the acronym **RULE**: **R**esist the righting reflex; **u**nderstand the patient’s individual concerns, motivations, and values; **l**isten to and empathetically reflect the patient’s thoughts; and **e**mpower the patient’s motivation and confidence in their ability to change. In addition, the therapist should accept the patient’s ambivalence toward the change and support the patient’s autonomy (35). Ambivalence is considered a normal component of the change process. According to Prochaska and DiClemente’s (60) transtheoretical model (TTM), change is a process consisting of six stages: (1) precontemplation, (2) contemplation, (3) preparation, (4) action, (5) maintenance, and (6) termination (Note: It is possible to return to earlier stages within the process). MI focuses on recognizing the current step of the patient’s change process and guiding them to move on to the next step. For example, in the contemplation step (2), the patient is ambivalent about whether to stop playing video games. In this case, the therapist can use *change talk* (35) to explore the discrepancies between the patient’s behavior and their values, e.g., “You said you enjoy playing video games, but you also mentioned social problems when participating in group activities.” The main principle of the MI-based therapeutic stance can be symbolized by the image of “dancing instead of wrestling” with the patient; that is, the patient is free to decide whether to reduce and control their usage behavior or to remain abstinent. We recommend that therapists attend basic MI training to be able to apply MI techniques during their sessions.

In addition, we recommend the following therapeutic attitudes: strive for a maximally resource-based therapy and validate the patients’ achievements where possible. Inquire with interest, but avoid judgments or confrontations about problematic behavior and resistance. Otherwise, you will quickly get into a psychotherapeutic transference and join the ranks of the critics (i.e., the parents, the school, etc.). This quickly leads online to the termination of therapy. Motivation for change sometimes takes time. This open and creative-affirmative attitude is especially important in short-term online therapy.

### 4.2. The manual: Basic structure and content of the sessions

The manual includes 12 webcam-based therapy sessions, each lasting 50 min. The content of the sessions is based on MI and CBT techniques (e.g., ACT). Except for Session 1, which focuses on diagnostics and relationship building (see below), each session is framed by a standardized beginning and conclusion/outlook. The therapist begins the session with a summary of the last session and an appreciation of the patient’s achievements. The therapist also summarizes the patient’s reasons for change and affirms the accuracy of their overview. Before presenting the content of the current session, the therapist asks the patient if they have anything else to share (e.g., important events since the last session). We recommend paying particular attention to the patient’s emotional level, as the level of distress may increase during the therapeutic process, e.g., due to withdrawal symptoms. Once all current concerns have been discussed, the therapist introduces the session’s specific content. Table 1 provides an overview of the content of each session and the associated worksheets.

**Table 1:** Therapy sessions and session content.

Session and topic	Content
Session 1*: Diagnostics	Diagnostic investigation of Internet use disorder (IUD; e.g., type and duration of Internet use, onset) and (relevant) comorbid syndromes. We recommend using standardized interviews for IUD and comorbidity diagnoses, e.g., the AICA-SKI-IUD or the DIPS (61, 62)
Session 2*: Building a relationship and anamnesis of Internet use	Further exploration of Internet usage behavior and life events associated with this behavior. Elaboration on the relationship between Internet use and life satisfaction (e.g., is Internet use a compensatory strategy for life dissatisfaction?) Optional: measurement of motivating factors, e.g., with the Internet-Use Expectancies Scale (63, 64). Implementation of self-observation in the form of daily logs and development of completion routines for the worksheets. The worksheets can be customized to the patient, e.g., a simple recording of usage time or a more complex recording of usage situations, associated thoughts, feelings, and body reactions (see: (62)). Active listening (35), clarification, and building the hypothesis of becoming addicted.
Session 3*: Identification of influencing factors	The addiction triangle model (65), adapted from IUD, is introduced as an individual model of the disorder, and influencing factors from the three areas are identified: environment—Internet (problematic types of Internet applications, e.g., video games, pornography usage, streaming)—person. Optional psychoeducational elements, depending on the patient’s individual situation, include: psychoeducation about addiction (e.g., addiction shift, compensation vs. gratification, abstinence vs. controlled behavior); procrastination; the impact of healthy routines and a regulated daily structure; and/or discussing health-related behaviors, such as eating, sleeping, social life, and physical activity.
Session 4: Setting (therapy) goals	Based on the influencing factors elaborated in Session 3’s definitions of three individual goals from each area (*worksheet*: “Addiction Triangle and Goals,” Appendix 1), encourage the patient to take an active role in decision-making to change their Internet usage behavior. We recommend defining goals according to the SMART principle (66):**s**pecific,**m**easurable,**a**ttractive,**r**ealistic, and**t**ime-bound. Previous studies have shown that self-efficacy is positively associated with short- and long-term treatment success (67–69). Optional elements, depending on the patient’s individual situation: Evaluation of self-observation and analysis of the patient’s daily structure using daily logs. Identify high-risk situations and unfavorable routines (e.g., boredom, conflicts, stress, habits, and automatisms). Analysis of thoughts, feelings, and body reactions associated with usage situations (see: (70)).
Session 5: Clarification of ambivalence and enhanced motivation to change	Raising awareness of ambivalences and promoting motivation to change using a cost–benefit analysis and elaborating concrete reasons for change. Writing down personal reasons and (long-term) benefits for a change in Internet use. *Worksheet*: We recommend using a 2×2 decision matrix to explore the pros and cons of change (35). Use MI techniques such as change talk and encourage the patient to take an active role in motivating change: *Example 1: Dealing with resistance* Active listening: Patient: “I do not think I’m using the internet in a problematic way.” Therapist: “You do not think that you use the internet in a problematic way.” Reflect both sides of the patient’s ambivalence: Patient: “I wish I could stop working on my internet use all the time.” Therapist: “You would like to stop working on your internet use. But, at the same time, there is a reason why you are working on it.” Reframing: Patient: “My wife criticizes me all the time.” Therapist: “That bothers you. At the same time, it means that your wife is worried about you. You must be very important to her.” Emphasizing autonomy: Patient: “I just do not want to change my internet use habits.” Therapist: “That is entirely your decision.” *Example 2: Eliciting change talk (DARN)* Therapist: D Desire “What are the things you would like to change?” “What do you dislike about your current situation?” A Ability “If you were to decide to change something, what would it be?” R Reason “What are good reasons for making a change?” N Need “Why might it be important?” “How serious is it?” “How urgent is it?”
Session 6: Developing alternative strategies and resources	Elaboration and collection of alternative routines, resources, hobbies, or coping strategies to change how people deal with themselves (e.g., through self-confidence), with other people (e.g., conflict), or with external circumstances (e.g., loneliness, boredom). Activating resources to explore alternative behavior strategies and increase self-efficacy.
Session 7: Exploring life values	Self-exploration of values and creating future perspectives using acceptance and commitment therapy (ACT). Guiding question: “What is important to me?” Development of concrete values in different areas of life (e.g., partnership, self, profession, health, and finances). We recommend using worksheets from the ACT treatment manual (71).
Session 8: Living with healthier Internet use	Empowerment and appreciation of the achievements by focusing on the individual benefits of the change. Developing a vision for living with healthier Internet use. Elaboration of high-risk situations for relapse and preparation of emergency cards. Guiding question: “What will I specifically do in high-risk situations?”
Session 9–Session 11	*Individual and optional sessions to reinforce the content discussed or use the optional session material.*
Session 12*: Reflecting on the therapy process and outlook	Reflecting on the therapy process and further acknowledging successes and changes. Discussing further therapy options, e.g., inpatient therapy, couple therapy, and psychotherapy for the comorbid disorder. Providing booster session(s). Goal-setting to develop a future perspective and plan the next steps (*worksheet:* “Tools and Future Perspective,” Appendix 2). Depending on the patient’s individual situation, develop relapse prevention strategies.
Optional session content	Psychoeducation, depending on the focus of the individual problem (e.g., addiction shift, abstinence vs. controlled behavior, individual model of the disorder, and relationships to comorbid disorders). Classification of Internet content, applications, or games into unproblematic (green), risky (yellow), and dangerous (red) (see (55)). Investigation of reasons for procrastination and development of helpful strategies against procrastination. We recommend using worksheets from a procrastination treatment manual (e.g., (72)). Work on other issues such as sexuality and partnership, finances, and learning plans. If applicable and necessary, we recommend referral to social counseling.

* Core sessions (standardized framework of intervention).

The basic structure of the intervention is flexible: The therapist can choose the sequence of sessions according to the individual patient’s needs. However, we recommend a standardized intervention framework (*core sessions*) that includes Sessions 1, 2, 3, and 12 (marked with an asterisk in Table 1). In addition, please note that the manual contains three optional sessions (Sessions 9, 10, and 11). These sessions are intended as buffers at any point in the treatment process, for example, when a patient’s current concerns fill an entire session, or the previous session’s content needs to be reevaluated or supplemented. In addition, the manual includes additional optional session content, such as delving into the topic of procrastination. Each session ends with a ritual question: “Why would it be good to use the internet in a less problematic way?” The question aims to focus on the vision of a positive future. By answering this question, the patient is encouraged to visualize the positive outcomes and feelings (e.g., freedom and closeness to others) they will experience in that positive future. If feasible, additional booster sessions can be scheduled to reflect on achievements and setbacks in the post-completion period (e.g., 3 months later). It has been our experience that patients are more likely to maintain their motivation to change if they have a set appointment after completion. In addition, based on our clinical experience, we recommend paying specific attention to social and environmental factors (e.g., employment status, ongoing pension proceedings, and pending applications) that may be associated with extensive Internet usage. Sometimes it may be appropriate to refer patients to social counseling services in addition to psychological treatment.

### 4.3. Example sessions

Below are three example sessions presented in more detail. Note that each session is framed by the standardized introduction and conclusion/outlook mentioned in the previous section.

#### 4.3.1. Session 2: Relationship building and Internet use anamnesis

*Session focus:* The main goal of the second session is to build a relationship with the patient and explore their current and lifelong Internet use.

1. *Opening the session:* The therapist warmly greets the patient and introduces the content of the current session. The therapist encourages the patient to let the therapist know at any time if issues need to be addressed. The therapist also explains that the sessions’ content can be handled in a flexible manner if necessary.
2. *Working on the session’s main focus:* The therapist conducts an anamnesis of Internet use. Table 2 provides an overview of possible questions.

**Table 2:** Possible questions for anamnesis of Internet use [inspired by (61)].

*Current Internet use:* What exactly do you do on the Internet that you find problematic or even addictive? (e.g., offline or online gaming, online pornography, online gambling, online shopping, social media use, Internet research, streaming) Exactly what types of online platforms or Internet applications do you use in a problematic way? What does that look like? Please describe. How many hours per day and how many days per week do you use this application (or applications) in a problematic way? *Development of problematic/addictive Internet use:* How many years ago did this problematic or addictive Internet use first occur? What were your life circumstances at that time? Did you experience any particular life event at that time? How would you describe the development of problematic/addictive Internet use up to the present? Have there been periods of abstinence? If so, how long were you abstinent? What strategies did you use to be abstinent, and were they helpful? What were your life circumstances at the time of abstinence? Did you experience any particular life event at that time? *Negative effects:* What are areas of your life negatively affected by problematic/addictive Internet use? Concerning area *X*: How strong are the negative effects, e.g., on a scale from 0 (no negative effects at all) to 10 (extreme negative effects)? How does problematic/addictive Internet use impair the following areas of your life? (a) school/education/job, (b) social contacts and leisure time, and (c) family and household duties, e.g., on a scale from 0 (no negative effects at all) to 10 (extreme negative effects)? *Need for change:* How badly do you want to change your problematic behavior?

The anamnesis questions can be combined with an exploration of the patient’s resources, for example, by emphasizing their existing abilities to change problematic behavior in the past successfully. This can motivate the patient, who usually focuses on the negative aspects of their behavior. In addition, it may be helpful to use validated scales to measure motivational factors, such as the Internet-Use Expectancies Scale (63, 64).

1. *Closing the session:* At the end of the session, we recommend giving the patient a worksheet to record their daily structure for a few days (e.g., a daily log sheet). It can be used to reflect on initial changes and positive effects in the following session. Then, the therapist closes the session by summarizing the most important steps and ideas of the past session and introducing the ritual question: “Why would it be good to use the internet in a less problematic way?”

#### 4.3.2. Session 3: Identification of influencing factors

*Session focus:* The third session aims to explore the causes of problematic Internet use.

1. *Opening the session:* The therapist begins the session with a summary of the last session, which includes an appreciation of the patient’s current achievements and the patient’s reasons for change, with a focus on reassurance or correction of their overview by asking the patient if they have anything significant to share (e.g., significant events since the last session). As the patient responds, the therapist pays particular attention to the patient’s emotional level and signs of stress, e.g., due to withdrawal symptoms. The therapist then gives an overview of the specific content of the current session.
2. *Working on the session’s main focus:* The therapist presents the worksheet “Addiction Triangle and Goals” (Appendix 1). The worksheet can be completed during the session in shared screen mode.
  - Step 1 (Psychoeducation): The therapist explains that problematic Internet use may be influenced by various factors: the (social) environment, the Internet application and its addictive features, and individual personality factors.
  - Step 2 (Influencing factors): Based on the triangle model (65), the therapist helps the patient identify the individual causes of their problematic Internet use. For many patients, this is a very motivating and relieving moment, as many understand for the first time that the solution to their problem can be found in different areas and does not just consist of forcing themselves to be more disciplined and self-controlled. In addition, it is usually a relief for patients to realize that multiple internal and external factors are involved in their usage behavior, which can be addressed independently during the intervention.
3. *Closing the session:* The therapist provides the patient with the worksheet that they have been working on and closes the session by summarizing the main steps and ideas of the past session and asking the following ritual question: “Why would it be good to use the internet in a less problematic way?”

#### 4.3.3. Session 12: Reflection on the therapy process and outlook

*Session focus:* The last session aims to reflect on the therapy process and acknowledge the successes and changes. It also aims to set further goals and work out the next steps to create a positive outlook for the future.

1. *Opening the session:* The therapist begins the session with a summary of the last session, including a credible appreciation of the patient’s current achievements and the patient’s reasons for change, with an emphasis on reassurance or correction of their overview, by asking the patient if they have anything important to share (e.g., crucial events since the last session). As the patient responds, the therapist focuses on the patient’s emotional level and signs of stress, e.g., due to withdrawal symptoms. Finally, the therapist provides an overview of the specific content of the current session.
2. *Working on the session’s main focus:* In the last session, the therapist focuses on celebrating the patient’s change process by highlighting and acknowledging the patient’s successes. For example, the patient might be asked, “What have you achieved in this short time?” “How satisfied are you with the current situation?” It could be helpful to look at worksheets from the beginning of the therapeutic process that show the difference between the patient’s current and previous Internet use to illustrate the patient’s progress. The last session is also about ensuring success. For example, the therapist could ask: “What would be important to keep in mind going forward?” “What kinds of tools are especially important for you to maintain the changes you have made?” To that end, the therapist presents the “Tools and Future Perspective” worksheet (Appendix 2). The worksheet can be completed during the session in shared screen mode.
  - Step 1: The therapist assists the patient in collecting the most important strategies for healthy Internet use and including them in their “toolbox” for their “emergency plan.”
  - Step 2: The patient is then asked to look into the future. What do they want to achieve in the next month, 6 months, and 12 months? Setting these milestones motivates the patient to think about how they can influence the future, promoting their self-efficacy and motivation.

Depending on the patient’s individual situation, the therapist may discuss other treatment options with the patient, such as inpatient therapy or self-help groups, or the therapist may offer booster sessions themselves in the near future. For some patients, it may be helpful to talk about techniques to prevent relapse. For example, the therapist might ask the patient:

- “What do you see as typical difficult situations that could lead to problematic internet use?” (e.g., stress at work or a conflict with a partner). “What are the early warning signs?” (e.g., restlessness, depressive mood)
- “What options do you have to avoid risky situations?”
- “If you experience a risky situation, what can you do?” “What has been helpful in the past?” “What people could support you?”

1. *Closing the session:* The therapist gives the patient the worksheet they have been working on and closes the session by summarizing the main steps and ideas of the past session and asking the ritual question, “Why would it be good to use the internet in a less problematic way?”

The effectiveness of the online-based manual was tested as part of the OMPRIS project (52). Key findings from the RCT on the efficacy of the intervention will be published elsewhere.

## 5. Discussion

### 5.1. Online-based therapy: Challenges and opportunities

Online-based therapy differs from traditional face-to-face therapy in several ways that present opportunities and challenges for both therapists and patients.

#### 5.1.1. Technical requirements

The online setting requires various technical prerequisites, such as digital and media skills among therapists and patients, to make an online-based session possible. A stable Internet connection is needed so that the interference of sound or images does not impair the flow of conversation. Technological devices such as a computer or tablet, webcam, and microphone are also required, which may incur an initial cost for patients. We recommend using a technical device with a sufficiently large screen to create a good working atmosphere. We, therefore, tend to advice against using a smartphone. To prevent persistent technical difficulties, scheduling sufficient time during the first consultation session is advisable to clarify any technical problems and check the requirements.

#### 5.1.2. Data security

Another challenge that online-based therapy faces is data security. The therapist must ensure that the platform used for the online session complies with the data protection regulations in force in their country. We recommend finding out about requirements and certified providers *via* the websites of national associations of statutory health insurance physicians (in Germany).^1^ Uncertainties that arise in patients regarding data security should be discussed at the beginning of therapy. Therefore, sufficient time must be devoted to this topic during the initial consultation session.

#### 5.1.3. Therapeutic relationship

In addition to the general conditions that must be clarified at the beginning of therapy, establishing contact *via* webcam also presents a challenge. In contrast to face-to-face therapy, two people meet in a digital space, which means that both the therapist and the patient have access to only part of each other’s information. Therefore, some nonverbal and para-verbal information may not be available, and making eye contact may be more challenging. The therapist must pay special attention to the patient in order to perceive and respond to all important information and signals and establish interpersonal closeness despite the distance (73). Regardless of the initial difficulties in establishing a relationship, it can be assumed that a sustainable working alliance can be established *via* the online format across different mental disorders. Patients and therapists perceive this working alliance as stable as face-to-face therapy (74–77). We recommend that the therapist create an open and productive working atmosphere from the beginning.

#### 5.1.4. Dealing with suicidality

A stable therapeutic relationship also allows for dealing with difficult situations online, such as suicidality or acute crises. However, some potentially relevant nonverbal and para-verbal information from the patient is missing in this setting. As mentioned above, we strongly recommend that the therapist actively asks about the patient’s current mood and any potential crises or concerns at the beginning of each session. In addition, the therapist should clarify the precise suicidal ideations of the patient and, in the case of acute suicidality, accompany the patient to local crisis treatment. Especially for patients with comorbid mental disorders, it is essential to speak transparently from the beginning about the procedure in case of acute suicidality or crises and to make agreements.

#### 5.1.5. Worksheets

Not only does the process of relationship building require a particular level of attention, but the treatment content must also be adapted with respect to face-to-face therapy. Worksheets must be adapted to digital versions (e.g., PDFs), which can be completed throughout the sessions. The therapist can fill out the worksheets, and the patient can see the worksheet *via* screen sharing or vice versa. Both approaches have different benefits and disadvantages. In the first case, for example, the therapist summarizes the patient’s ideas in their own words, allowing the patient to clarify their understanding. The therapist then accompanies the patient rather than observing the patient’s solutions. In this case, however, the patient is more passive than in the second approach, where the patient actively contributes to the worksheet preparation. In addition, the therapist should determine whether the worksheets can be sent directly *via* the video platform or by email before or after the sessions.

#### 5.1.6. Setting

Because the sessions do not take place in the therapist’s office, the patient can choose the setting for online therapy. Thus, they can participate from almost anywhere. This aspect poses another challenge because the patient may be in a (public) place that is not conducive to therapeutic conversations. As a result, privacy and data protection may be compromised, and the patient may be inhibited from talking openly about difficult topics. Even in protected spaces, such as the patient’s home, it is necessary to deal with distracting background noise, the presence of other family members, and other environmental factors (e.g., a television in the background of the patient’s room). It is advisable to clarify this aspect right at the beginning and find solutions for potentially obstructive surrounding factors so that they do not have an ongoing influence on the therapeutic process (73).

On the other hand, a private setting can be beneficial for the therapeutic process. In this way, the therapist gains direct and unfiltered insights into the reality of the patient’s life and environment. In our practical experience, we have recognized that the environment contains important information for creating working hypotheses. For example, the presence of family members who constantly interrupt the conversation might indicate a lack of privacy or point to a place of retreat where the patient’s previous solution was using the Internet. The therapist may also actively gain insight into the patient’s living space, for example, by asking to be shown the patient’s computer corner. When taken together, the therapist can perceive environmental information, derive hypotheses, and work on them together with the patient if the hypothesis is coherent, which is a clear advantage over face-to-face therapy.

Changing environmental factors (e.g., an untidy apartment or inconvenient computer placement) can also be done much more quickly in an online-based setting. Moreover, the private setting of the sessions can create a closeness to the patient and the reality of their life, making it easier to integrate insights and resources into everyday life. For example, the therapist can advise the patient to make a written record of important session content and keep it at the patient’s home. This ensures a simple and quick transfer, and the patient can continue working on the topics beyond the sessions.

#### 5.1.7. Accessibility and reachability

A major advantage of the setting is its independence in terms of location. People who, for various reasons, could not reach a therapeutic practice have access to adequate treatment, e.g., people living in rural areas. This is of particular interest for conditions with few specialized health care services, such as IUDs. It is also accessible to people with mobility restrictions and limited time capacities, e.g., due to work or family commitments. In addition, therapy can be easily integrated into daily life and offers more flexibility in appointments and session frequency than in-person sessions. Especially during the COVID-19 pandemic, it has been important to avoid face-to-face contact to prevent infection and protect at-risk patients. Online-based therapy also allows individuals who are in quarantine or are unable to reach an in-person treatment site because of other circumstances to receive treatment (78, 79). Consistent with scientific evidence, we have observed that patients report an increase in preexisting IUD symptoms as well as other mental health conditions, such as depression or anxiety disorders, as a result of the pandemic (80–82). Despite the pandemic situation, online-based therapy allows for low-threshold therapy for patients suffering from comorbid anxiety disorders or very low levels of functioning that prevent them from seeking face-to-face treatment (77, 83). Low-threshold services are of particular importance in reaching people suffering from SUDs (84–86) and behavioral addictions (87), who regularly have low intrinsic motivation and are ambivalent about changing their behavior (88). Moreover, the sense of distance between therapist and patient may even facilitate talking about shameful topics, such as the use of online pornography.

#### 5.1.8. Cost-effectiveness

In terms of national health systems, it is also beneficial that online therapies are more cost-effective than face-to-face therapies due to lower operating costs and reduced therapist costs (83, 89). In addition, it could be hypothesized that individuals will seek treatment at an earlier stage of the disease due to the enhanced reachability and accessibility of the setting, resulting in lower long-term costs for the health care system. Social anxiety is a significant comorbidity of IUD (90). People suffering from social anxiety are more likely to use other means of contact than face-to-face contact, leading to earlier treatment initiation for this specific group. This leads to a shortened chronification and, thus, a much higher treatment success rate, as the treatment may begin much earlier for people suffering from IUD and social anxiety.

## 6. Conclusion

The treatment of IUDs is of increasing importance, but specialized therapies are rarely available for those affected. To our knowledge, this short-term manual is the first designed for online-based therapy of IUDs. We have combined different forms of therapy (MI, CBT, and ACT), whose effectiveness has already been clinically proven, especially in the context of SUDs and addictive behaviors, and adapted them for online use. On this basis and through the possibility of individualization, it is possible to work with a wide range of patients, regardless of the severity of the disorder. In this way, the manual’s content can also be used preventively to reduce the development of addiction symptoms and chronification, which can relieve the burden on the health care system in the long term. Furthermore, especially in the COVID-19 pandemic, the relevance of alternative forms of therapy became evident, so the low-threshold character of the online-based approach should also be emphasized here. Thus, patients unable to attend regular face-to-face therapy for personal, pathological, or environmental reasons are given access to therapy. We view the accessibility and reachability of the online-based treatment approach for IUDs as a promising opportunity to fill an important gap and reach the widest possible patient population.

From our point of view, low-threshold telemedicine interventions can bridge the gap between the analog healthcare system and affected people who do not have the opportunity or see the need to undergo therapeutic treatment. Specifically, people who overuse the Internet can be directly reached where they are. Webcam-based therapy can provide a bridge back to analog life. We, therefore, see telemedicine as a complement to, but not a replacement for, face-to-face therapy.

## 7. Practical take-home messages

- Therapists and patients must have the technical prerequisites and also the motivation to do therapy *via* webcam. This is often less of a problem with the patient group, as they have an affinity for the Internet anyway. Psychotherapists, however, have not learned how to provide therapy online in their training. In this case, we recommend taking further training on the topic and learning about the positive effects of online therapy. This can open the mindset of the therapists and make a good process possible. Only if psychotherapists are convinced that their online therapy works, it will be a success. The technical requirements are often quickly installed and do not cost much. Certified providers often offer free user licenses for individual workstations.
- Psychotherapists need to understand in advance that relationship building feels a little different in online therapy. On the one hand, the setting often leads patients to address even shameful topics more quickly; on the other hand, patients are out of contact more quickly when confronted and fixated on the problem. Therefore, a very resource-oriented and motivating therapeutic attitude is helpful. In particular, one should avoid getting into a scuffle about “too much Internet use” (parent transference).
- Psychotherapists must have a concept in advance of how to deal with patients’ psychological crises and what arrangements are made in advance. These should already be discussed at the beginning. In particular, it should be stated that the online therapist has the option of contacting local help services (social psychiatric service, police if necessary) in the event of an emergency (active suicidal tendencies). If it is not possible to assess suicidality sufficiently well *via* webcam, the indication for online therapy should be questioned.
- Collaborative editing of worksheets, usage logs, and therapeutic charts is a bit more difficult online. There is a technical solution of a shared whiteboard where therapeutic materials can be completed and discussed together. Therapists should not be afraid to try different modes of using therapeutic materials here (e.g., offline pdf files vs. online whiteboard).
- Patients with IUD often appreciate the possibility of online therapy. It is possible to build a bridge into analog therapeutic settings. This should be taken into account when setting goals.

## Data availability statement

The original contributions presented in the study are included in the article/supplementary material, further inquiries can be directed to the corresponding author.

## Author contributions

MP: conceptualization and writing-original draft. BG: writing-original draft. LC: literature review and research assistance. LB: conceptualization and review and editing. BW: study supervision and review and editing. MD: review and editing. SH: study supervision and review and editing. JD-H: conceptualization, study supervision and review and editing. All authors contributed to the article and approved the submitted version.

## Funding

The project was funded by the German Innovation Fund of Germany’s Federal Joint Committee (01VSF18043).

## Conflict of interest

The authors declare that the research was conducted in the absence of any commercial or financial relationships that could be construed as a potential conflict of interest.

## Publisher’s note

All claims expressed in this article are solely those of the authors and do not necessarily represent those of their affiliated organizations, or those of the publisher, the editors and the reviewers. Any product that may be evaluated in this article, or claim that may be made by its manufacturer, is not guaranteed or endorsed by the publisher.

## Figures and Tables

**Figure 1:**
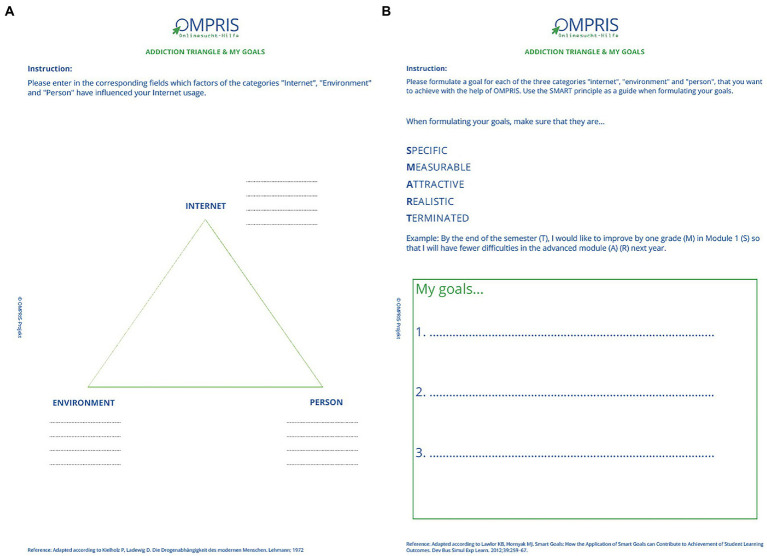
**(A, B)** Addiction triangle and goals.

**Figure 1:**
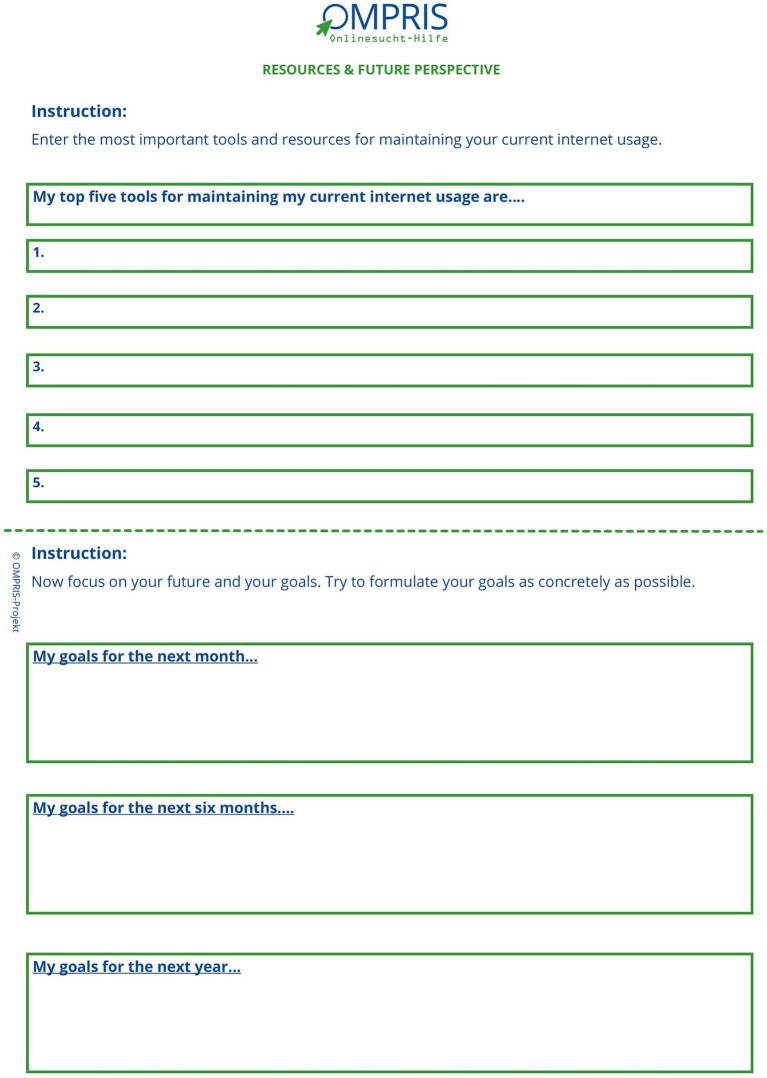
Tools and future perspective.

## Footnotes

^1^ https://www.kbv.de/html/videosprechstunde.php
